# 

**Published:** 1894-07

**Authors:** 


### CONTENTS—JULY, 1894.—VOL. X., NO. 1.

ORIGINAL COMMUNICATIONS—
A Review of Dr. Wallace and the Railway Surgeons, on Spinal Concussions. R. M. Swearingen, M.D., Austin . . . . . . . . . . . . . 1
Some remarks on the Ætiology, Pathology and Treatment of Malarial Hæmatinuria. T. F. Oates, M. D., Mexia . . . . . . . . . . . . . 11
Endometritis. T. J. Wagley, M. D., Cleburne . . . . . . . . . . . . 15
ABSTRACTS OF CURRENT MEDICAL LITERATURE—
Department of Practice of Medicine. Edited by Prof. A. J. Smith, M. D., Galveston. . . . . . . . . . . . . . . . . . . . . . . . . . . 20
Department of Gynecology. Edited by Prof. Wm. Keiller, F. R. C. S., Ed., Galveston . . . . . . . . . . . . . . . . . . . . . . . . . . . 24
SOCIETY NOTES—
Call for Organization of a District Society . . . . . . . . . . . . . 25
Medical Board Organized. . . . . . . . . . . . . . . . . . . . . . . . 26
EDITORIAL—
A Spook that Will Not Down. . . . . . . . . . . . . . . . . . . . . . 27
Death of Prof. Briggs. . . . . . . . . . . . . . . . . . . . . . . . . 29
Prof. Sim. . . . . . . . . . . . . . . . . . . . . . . . . . . . . . . 30
MEDICAL NEWS AND MISCELLANY—
Many Interesting Paragraphs . . . . . . . . . . . . . . . . . . . . . 3
Retrospective; Prospective. . . . . . . . . . . . . . . . . . . . . . 33
BOOK NOTICES— . . . . . . . . . . . . . . . . . . . . . . . . . . . . 35
PUBLISHERS' NOTES . . . . . . . . . . . . . . . . . . . . . . . . . . 39

